# Long-term outcomes of spinal cord stimulation for refractory phantom limb pain

**DOI:** 10.3389/fpain.2026.1955515

**Published:** 2026-09-07

**Authors:** Long Ma, Yunjian Huang, Shiyun Xu, Guang Lu, Hongwei Zhu

**Affiliations:** 1 Department of Pain Medicine, Sanya Central Hospital (The Third People's Hospital of Hainan Province), Sanya, Hainan, China; 2 Department of Neurosurgery, Xuanwu Hospital, Capital Medical University, Beijing, China

**Keywords:** clinical outcomes, device explantation, neuropathic pain, phantom limb pain, spinal cord stimulation

## Abstract

**Objectives:**

Phantom limb pain (PLP) is a disabling post-amputation neuropathic pain condition, and evidence regarding the long-term effectiveness of spinal cord stimulation (SCS) remains limited. This study evaluated trial stimulation outcomes, pain trajectories, device retention, explantation, and device-related complications in patients with refractory PLP.

**Methods:**

We retrospectively reviewed 28 patients with refractory PLP who underwent SCS trial stimulation at our center between June 2010 and September 2023. Baseline characteristics, procedural data, Numeric Rating Scale (NRS) scores, progression to permanent implantation, revision or replacement procedures, and device explantation were recorded. Changes in NRS scores from baseline were assessed, and exploratory regression analyses were performed.

**Results:**

Eighteen patients (64%) met the criteria for trial success and proceeded to permanent SCS implantation. In an exploratory analysis, a higher baseline NRS score was associated with trial success. Among the 18 patients with permanent implants, the median follow-up was 78 months. Five SCS systems (28%) were explanted: three because of inadequate pain relief and two after battery depletion without replacement. In a *post hoc* analysis of the 13 patients who retained their devices at the last available follow-up, the mean NRS score decreased from 8.9 ± 0.8 at baseline to 2.8 ± 0.7 after trial stimulation and was 4.8 ± 0.6 at 1 year, 5.6 ± 1.0 at 2 years, and 6.2 ± 1.1 at the last available follow-up. Three patients underwent planned IPG replacement because of battery depletion, and two underwent unplanned device-related revision procedures.

**Conclusions:**

SCS provided substantial short-term pain relief in selected patients with refractory PLP. However, the magnitude of pain relief diminished over time, and device explantation occurred in a substantial minority of patients, underscoring the need for careful patient selection, long-term follow-up, and device management.

## 1 Introduction

Phantom limb pain (PLP) is a common and disabling form of post-amputation neuropathic pain characterized by painful sensations perceived in the absent limb. Although reported prevalence estimates vary across populations and assessment methods, recent systematic reviews indicate that PLP affects approximately 6.7%–88.1% of individuals after amputation, with a pooled prevalence of 64% and a lifetime prevalence of up to 87% (1–3). PLP is frequently accompanied by residual limb pain, nonpainful phantom sensations, emotional distress, impaired prosthesis use, sleep disturbances, and reduced quality of life (3, 4). Several clinical factors, including pre-amputation pain, residual limb pain, lower-limb amputation, a more proximal amputation level, and nonpainful phantom sensations, have been associated with the development and persistence of PLP (1, 5).

Because post-amputation pain can arise from different sources, it is important to distinguish PLP from pain originating in the residual limb. Residual limb pain is localized to the remaining limb and may result from local nociceptive or mechanical causes, including painful neuroma, which represents a peripheral neuropathic pain condition arising from injured or transected nerves. In contrast, PLP is perceived in the absent portion of the limb and is considered a neuropathic pain syndrome; these pain conditions may coexist in the same patient. The pathophysiological mechanisms underlying PLP are complex and remain incompletely understood. Current evidence supports a multifactorial model involving peripheral mechanisms, such as neuroma formation and ectopic neural discharge; spinal mechanisms, including central sensitization and altered dorsal horn processing; and supraspinal mechanisms, including maladaptive cortical reorganization, disrupted body representation, and abnormal sensorimotor integration (3, 4, 6, 7). This mechanistic heterogeneity may partly explain the challenges associated with treating PLP and the marked interindividual variability in treatment response.

Current treatments for PLP include pharmacotherapy, rehabilitative and psychological interventions, sensorimotor therapies, and peripheral nerve procedures. However, evidence supporting many of these treatments remains inconsistent, and long-term or durable pain relief has not been established for a substantial proportion of patients (8–11). For patients with severe, refractory PLP, implantable neuromodulation may be considered after conservative treatments have failed to provide adequate relief.

Spinal cord stimulation (SCS) is an established neuromodulation therapy for selected patients with chronic, refractory neuropathic pain (12–14). Its analgesic mechanisms are complex and remain incompletely understood. Beyond the classical gate control theory, SCS may modulate descending inhibitory pathways, including noradrenergic and serotonergic signaling. Studies of high-frequency and burst stimulation have suggested additional mechanisms, including attenuation of hyperexcitability in dorsal horn wide-dynamic-range neurons and modulation of small-diameter nerve fibers (15). Previous studies suggest that SCS may provide clinically meaningful pain relief in selected patients with PLP; however, the available evidence is largely limited to case reports, small retrospective case series, and heterogeneous neuromodulation cohorts (16–19). Moreover, the long-term durability of analgesia, device retention and explantation rates, and factors associated with trial success or device explantation remain insufficiently characterized.

Therefore, this retrospective cohort study aimed to evaluate the long-term clinical outcomes of SCS in patients with refractory PLP. Specifically, we assessed trial stimulation outcomes, longitudinal changes in pain intensity, device retention and explantation, device-related complications and revision procedures, and clinical factors associated with trial response and device explantation.

## 2 Methods

### 2.1 Study design and participants

This retrospective cohort study was conducted at Xuanwu Hospital, Capital Medical University, to evaluate the long-term clinical outcomes of SCS in patients with refractory PLP. We reviewed the medical records of patients who underwent SCS trial stimulation between June 2010 and September 2023. Patients were eligible if they met the following criteria: (1) a clinical diagnosis of PLP after limb amputation; (2) PLP persisting for at least 3 months despite conservative treatment; (3) age ≥18 years at the time of trial lead implantation; and (4) sufficient baseline and follow-up data for analysis. Patients were excluded if they had previously undergone SCS implantation at another institution, had an uncontrolled severe psychiatric disorder, or had pain predominantly attributable to residual limb pathology rather than PLP. The study was approved by the Ethics Committee of Xuanwu Hospital, Capital Medical University. The requirement for written informed consent was waived by the Ethics Committee because of the retrospective nature of the study.

### 2.2 Surgical procedure

SCS leads were placed in the epidural space either percutaneously or surgically through a laminotomy. Surgical paddle leads (Resume II 3587A, Specify 3,998, and Specify 5-6-5 39,565; Medtronic, Dublin, Ireland) were implanted under monitored anesthesia care with intravenous sedation. Percutaneous leads (1 × 8 Standard Lead 3,777; Medtronic, Dublin, Ireland) were implanted under local anesthesia to permit intraoperative assessment of paresthesia coverage. The choice between a surgical paddle lead and a percutaneous lead was individualized according to anatomical feasibility, the anticipated stimulation coverage, and the treating surgeon's clinical judgment rather than a predefined study protocol. Surgical paddle leads, although requiring laminotomy, were generally used when stable epidural positioning and durable paresthesia coverage were prioritized. Percutaneous leads were used when a less invasive approach was considered feasible and permitted intraoperative paresthesia mapping under local anesthesia. Because lead selection was not randomized, comparisons according to lead type were considered exploratory.

The vertebral level of lead placement was selected according to the anatomical distribution of PLP. Cervical leads were used for upper-limb PLP, whereas thoracic leads were used for lower-limb PLP. Lead position was adjusted intraoperatively to achieve adequate paresthesia coverage of the painful phantom territory. All patients underwent a trial stimulation period with a median duration of 7 days (range, 3–14 days). Trial stimulation was considered successful when patients achieved a ≥ 50% reduction in NRS score from baseline together with adequate and tolerable paresthesia coverage of the painful phantom territory. Patients who met these criteria and wished to continue SCS therapy proceeded to IPG implantation. In these patients, the trial lead was retained and connected to the IPG, thereby avoiding lead replacement.

### 2.3 Clinical evaluation

Data on SCS treatment, revision or replacement procedures, device-related complications, and device explantation were extracted from the medical records. Baseline variables included age, sex, pain location, pain duration, Numeric Rating Scale (NRS) score, presence of residual limb pain, cause of amputation, and lead type. Follow-up data were collected during routine clinical care and supplemented by medical record review and telephone follow-up. The study follow-up period ended in May 2026.

### 2.4 Statistical analysis

Statistical analyses were performed using IBM SPSS Statistics version 27.0 (IBM Corp., Armonk, NY, USA). Continuous variables were presented as the mean ± standard deviation (SD) or median (range), as appropriate, and categorical variables as numbers and percentages. Changes in NRS scores at each follow-up assessment were compared with baseline using the Wilcoxon signed-rank test. Trial success was defined as described above. Logistic regression analysis was used to explore factors associated with successful trial stimulation. Cox proportional hazards regression analysis was used to explore factors associated with device explantation among patients who underwent permanent SCS implantation. Given the small sample size and limited number of outcome events, all regression analyses were considered exploratory. All tests were two-sided, and a *P* value <0.05 was considered statistically significant.

## 3 Results

### 3.1 Patient characteristics

Among the 28 patients, the mean age at trial stimulation was 56.5 ± 10.7 years, and 8 patients (29%) were female. The median pain duration before SCS was 3.0 years (range, 1–38 years), and the mean baseline NRS score was 8.5 ± 0.8. Eleven patients (39%) had upper-limb PLP, whereas 17 (61%) had lower-limb PLP. Residual limb pain was present in 9 patients (32%). The causes of amputation were trauma in 15 patients (54%), vascular disease in 4 (14%), tumor in 5 (18%), and infection in 4 (14%).

### 3.2 Trial stimulation

**Figure 1:**
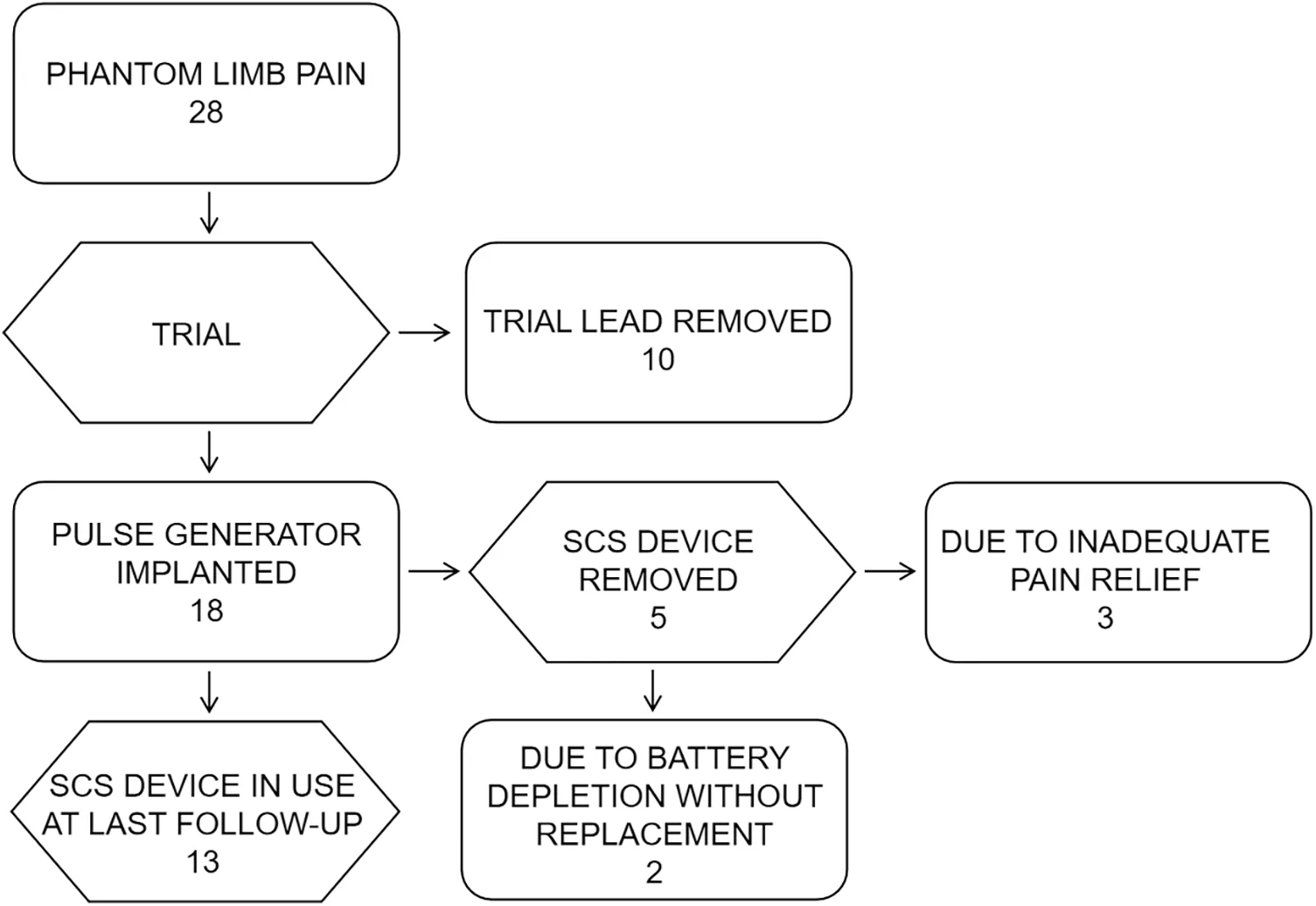
Flowchart of 28 patients with phantom limb pain who underwent spinal cord stimulation from June 2010 to September 2023.

All 28 patients underwent trial stimulation. Eighteen patients (64%) met the predefined criteria for a successful trial, defined as a ≥ 50% reduction in NRS score from baseline together with adequate and tolerable paresthesia coverage of the painful phantom territory, and subsequently underwent permanent SCS implantation. The remaining 10 patients (36%) did not achieve adequate pain relief during the trial and had their trial leads removed (Figure 1). Surgical paddle leads were used in 21 patients (75%), whereas percutaneous leads were used in 7 (25%).

In the exploratory logistic regression analysis, a higher baseline NRS score was associated with trial success (OR, 5.57; 95% CI, 1.22–25.45; *P* = 0.03). Age, sex, pain location, pain duration, presence of residual limb pain, cause of amputation, and lead type were not significantly associated with trial success (Table 1).

**Table 1:** Patient characteristics and exploratory regression analyses of factors associated with successful trial stimulation and SCS explantation.

				Permanent SCS implanted *n* = 18			
	Trial only *n* = 10			SCS explanted *n* = 5			SCS in use at the end of follow-up *n* = 13
Variable	*n* (%)	OR (95% CI)^a^	*P* value	*n* (%)	HR (95% CI)^b^	*P* value	*n* (%)
Age, mean ± SD	58.4 ± 11.3	0.97 (0.90–1.05)	0.47	52.6 ± 10.3	0.95 (0.86–1.05)	0.33	56.5 ± 10.8
Sex							
Female	4 (40)	1		1 (20)	1		3 (23)
Male	6 (60)	2.33 (0.43–12.57)	0.40	4 (80)	1.11 (0.12–10.01)	0.92	10 (77)
Pain location							
Upper limb	3 (30)	1		5 (100)	1		3 (23)
Lower limb	7 (70)	0.54 (0.10–2.77)	0.69	0 (0)	n.d.	n.d.	10 (77)
Pain duration, median (range)	3.5 (1–18)	1.02 (0.93–1.12)	0.66	3.0 (2–6)	0.90 (0.72–1.13)	0.36	3.0 (1–38)
NRS score, mean ± SD	8.0 ± 0.7	5.57 (1.22–25.45)	**0**.**03**	8.6 ± 0.9	0.71 (0.20–2.47)	0.59	8.9 ± 0.8
Presence of residual limb pain	4 (40)	0.58 (0.11–2.95)	0.68	1 (20)	0.43 (0.05–3.97)	0.46	4 (31)
Cause of amputation							
Trauma	5 (50)	1		2 (40)	1		8 (62)
Vascular disease	3 (30)	0.17 (0.01–2.04)	0.26	0 (0)	n.d.	n.d.	1 (8)
Tumor	1 (10)	2.00 (0.17–22.95)	1.00	2 (40)	2.41 (0.33–17.61)	0.39	2 (15)
Infection	1 (10)	1.50 (0.12–18.36)	1.00	1 (20)	1.16 (0.10–13.26)	0.90	2 (15)
Lead type							
Surgical	6 (60)	1		3 (60)	1		12 (92)
Percutaneous	4 (40)	0.30 (0.05–1.76)	0.21	2 (40)	6.30 (0.88–45.00)	0.07	1 (8)

Data are presented as mean ± SD, median (range).

SCS, spinal cord stimulation. n.d., not defined.

Bold values indicate statistically significant associations in the regression analyses (*P* < 0.05).

a Odds ratios were calculated using exploratory logistic regression analysis for successful trial stimulation.

b Hazard ratios were calculated using exploratory Cox regression analysis for SCS explantation.

### 3.3 Explantation rate

Among the 18 patients who underwent permanent SCS implantation after the trial period, the median follow-up was 78 months (range, 32–190 months), corresponding to a total of 1,710 patient-months of follow-up. During follow-up, 5 SCS systems (28%) were explanted: 3 (60%) because of inadequate pain relief and 2 (40%) after battery depletion without replacement. Of the latter two patients, one declined IPG replacement because of financial constraints, whereas the other remained satisfied with pain control after stimulation ceased. The remaining 13 patients (72%) retained their SCS systems and continued SCS therapy at the last available follow-up.

Of the 3 patients with percutaneous leads, 2 (67%) underwent device explantation, compared with 3 of the 15 patients (20%) with surgical paddle leads. Among the 5 patients whose devices were explanted, the median time to explantation was 36 months (range, 21–78 months). In addition, 3 patients underwent planned IPG replacement because of battery depletion at 55, 58, and 62 months after implantation, respectively, and continued SCS therapy thereafter.

In the exploratory Cox proportional hazards regression analysis, none of the examined variables, including age, sex, pain location, pain duration, baseline NRS score, presence of residual limb pain, cause of amputation, and lead type, was significantly associated with device explantation. Given the small sample size and limited number of explantation events, these findings should be interpreted cautiously (Table 1).

### 3.4 Pain outcomes

**Figure 2:**
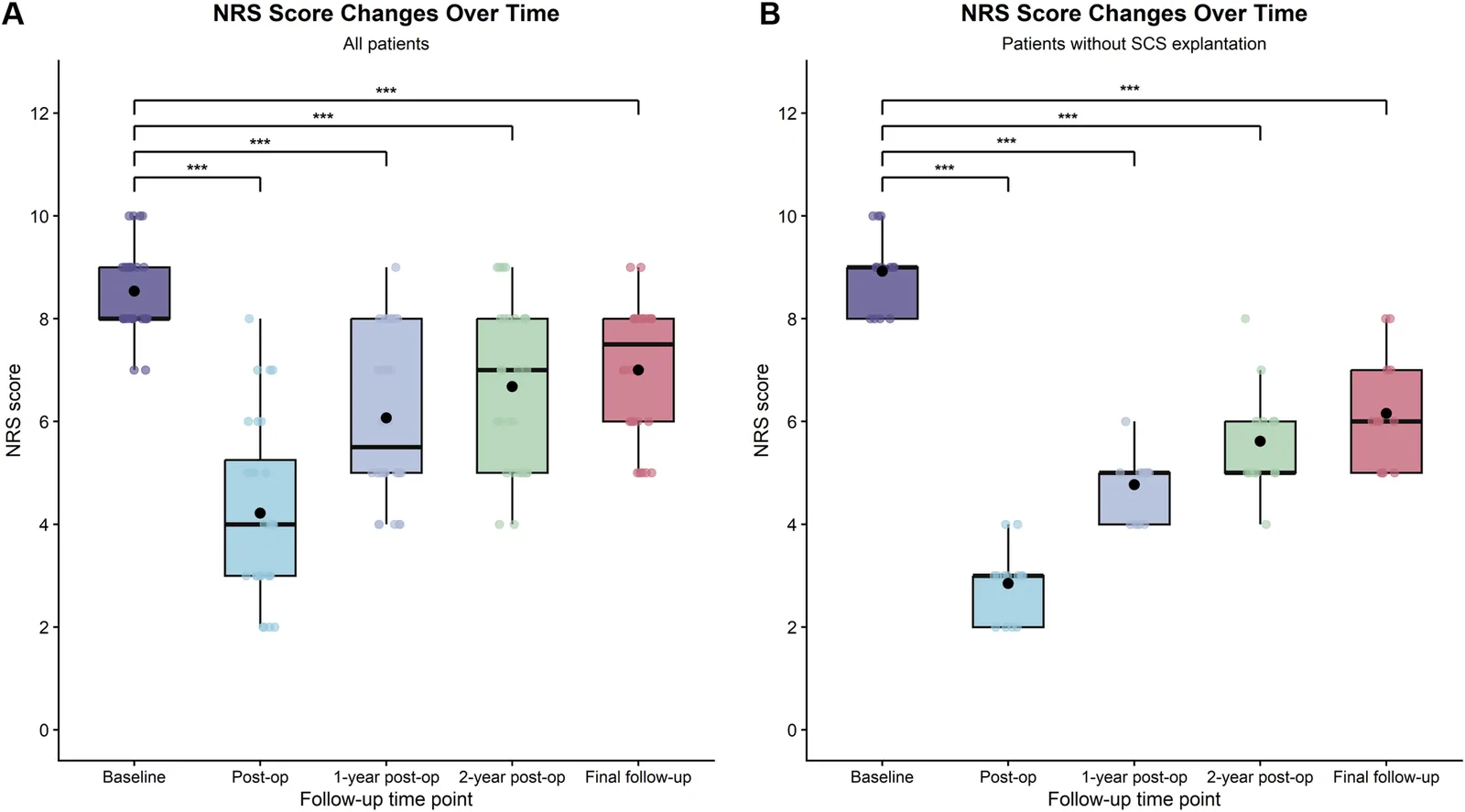
Longitudinal changes in pain intensity following spinal cord stimulation for phantom limb pain. **(A)**, Changes in NRS scores in the overall cohort. **(B)**, Changes in NRS scores in patients who retained their SCS devices at the last available follow-up. NRS scores decreased significantly after trial stimulation and remained significantly lower than baseline at the 1-year, 2-year, and last available follow-up assessments, although partial recurrence of pain was observed over time. Follow-up was completed in May 2026. ***P < 0.001 versus baseline. In the figure, ‘Post-op' denotes the assessment performed at the end of the trial stimulation period rather than after permanent IPG implantation. IPG, implantable pulse generator; NRS, Numeric Rating Scale; SCS, spinal cord stimulation.

Pain intensity was assessed using the NRS at baseline, at the end of trial stimulation, at 1 year, at 2 years, and at the last available follow-up (Figure 2). In the overall cohort of 28 patients, the mean NRS score decreased from 8.5 ± 0.8 at baseline to 4.2 ± 1.8 at the end of trial stimulation and subsequently increased to 6.1 ± 1.6 at 1 year, 6.7 ± 1.6 at 2 years, and 7.0 ± 1.3 at the last available follow-up. NRS scores remained significantly lower than baseline at all assessment time points (all *P* < 0.001). A ≥ 50% reduction in NRS score was achieved by 18 patients (64%) at the end of trial stimulation, 6 (21%) at 1 year, 4 (14%) at 2 years, and 1 (4%) at the last available follow-up. However, these descriptive results included patients with different subsequent treatment and device statuses and therefore should not be interpreted as representing the long-term effect of ongoing active SCS therapy.

Among the 13 patients who retained their SCS systems at the last available follow-up, the mean NRS score decreased from 8.9 ± 0.8 at baseline to 2.8 ± 0.7 at the end of trial stimulation and was 4.8 ± 0.6 at 1 year, 5.6 ± 1.0 at 2 years, and 6.2 ± 1.1 at the last available follow-up. NRS scores remained significantly lower than baseline at all assessment time points in this subgroup (all *P* < 0.001). A ≥ 50% reduction in NRS score was achieved by 13 patients (100%) at the end of trial stimulation, 5 (38%) at 1 year, 3 (23%) at 2 years, and 1 (8%) at the last available follow-up. This *post hoc* analysis was restricted to patients who retained their devices at the last available follow-up and was therefore subject to survivor bias.

### 3.5 Device-related complications and revisions

During follow-up, two device-related revision procedures were performed among the 18 patients with permanent SCS implants: one (6%) patient underwent IPG replacement because of IPG malfunction, and one (6%) underwent lead repositioning because of lead migration. No infections, neurological complications, local pain at the IPG site, or extension cable malfunctions were documented during follow-up.

## 4 Discussion

In this retrospective cohort of 28 patients with refractory PLP, SCS trial stimulation produced substantial short-term pain relief, and 18 patients (64%) met the predefined criteria for trial success and proceeded to permanent SCS implantation. Among patients with permanent implants, the median follow-up was 78 months. Five of the 18 SCS systems (28%) were explanted, whereas 13 patients (72%) retained their devices at the last available follow-up. For clinical context, these rates were broadly comparable with those reported in a long-term cohort of patients with persistent spinal pain syndrome type II, in which 78% of patients proceeded to permanent implantation after trial stimulation, approximately 26% of implanted systems were subsequently explanted, and approximately 74% remained in use during long-term follow-up. However, this comparison should be interpreted cautiously because of differences in patient populations and study design (20). Pain intensity decreased markedly at the end of trial stimulation but gradually increased thereafter, indicating attenuation of analgesic benefit over time. These findings suggest that SCS may provide clinically meaningful analgesia in selected patients with refractory PLP, although the durability of pain relief varies substantially among patients.

The short-term response observed in our cohort is consistent with previous reports suggesting that SCS may reduce PLP in appropriately selected patients. Early studies of dorsal column stimulation in amputees documented sustained pain reduction in some patients, although outcomes were heterogeneous and therapeutic efficacy often diminished over time (21, 22). Katayama et al. compared SCS, thalamic deep brain stimulation, and motor cortex stimulation in patients with PLP and found that SCS achieved satisfactory long-term pain control in a subset of patients, although treatment responses were not universal (23). Similarly, Viswanathan et al. reported that SCS may be beneficial for medically refractory PLP in carefully selected patients (24). More recent reviews have also highlighted the potential role of SCS in PLP while emphasizing the low quality of the available evidence and the need for longer-term outcome data (17, 25). Our study adds to this literature by providing a relatively long follow-up period and reporting both longitudinal pain trajectories and device explantation outcomes.

Although NRS scores remained significantly lower than baseline at all assessment time points, the magnitude of pain relief diminished over time. In the overall cohort, the mean NRS score decreased from 8.5 at baseline to 4.2 at the end of trial stimulation and subsequently increased to 7.0 at the last available follow-up. A similar temporal pattern was observed in the device-retention subgroup. However, the overall cohort included patients with different subsequent treatment and device statuses, whereas the device-retention subgroup was subject to survivor bias; therefore, these findings should be interpreted as descriptive pain trajectories rather than definitive evidence of progressive loss of SCS efficacy. A decline in analgesic benefit over time has been reported in SCS therapy and is sometimes described as tolerance or habituation. In contrast, Mekhail et al. reported durable reductions in pain intensity with evoked compound action potential–controlled closed-loop SCS, with no evidence of diminished therapeutic effect through 36 months of follow-up (26). Although that study was not specific to PLP, its findings suggest that closed-loop stimulation may help maintain analgesic efficacy over time.

The trial-to-permanent implantation rate in our study was 64%. Although this rate may be lower than those reported in some SCS cohorts involving other neuropathic pain conditions, direct comparisons are limited by differences in patient populations, indications, trial protocols, and definitions of trial success. Current consensus recommendations emphasize careful patient selection, psychological assessment, realistic expectation setting, and trial stimulation before permanent SCS implantation (13). In the exploratory logistic regression analysis, a higher baseline NRS score was associated with successful trial stimulation. However, this finding should be interpreted cautiously because of the small sample size and wide confidence interval. One possible explanation is that patients with greater baseline pain severity had more opportunity to achieve a clinically perceptible reduction in pain during the trial period. This association may also reflect residual confounding or selection bias inherent in the retrospective study design.

Device explantation occurred in 5 of the 18 patients (28%) who underwent permanent SCS implantation. The reasons for explantation were inadequate pain relief in 3 patients and battery depletion without device replacement in 2. This pattern is consistent with the broader SCS literature, in which loss of efficacy or inadequate pain relief is among the most frequently reported reasons for explantation (27, 28). In a long-term cohort of patients with complex regional pain syndrome, Hoikkanen et al. reported a 30% explantation rate over a median follow-up of 8 years, primarily because of insufficient pain relief (29). Long-term studies of SCS and dorsal root ganglion stimulation have likewise shown that device explantation remains a clinically relevant issue in chronic pain neuromodulation (27). The explantation rate observed in our cohort therefore falls within the range reported in other chronic neuropathic pain populations, although direct comparisons are limited by differences in indication, stimulation technology, follow-up duration, and definitions of explantation.

Emerging neuromodulation technologies may expand future treatment options for PLP. Sensory-restorative closed-loop SCS has shown promising results in restoring somatosensory feedback and reducing PLP in patients with lower-limb amputation (30). High-frequency SCS has also been described in selected patients with PLP and may improve pain control and prosthesis use, although the supporting evidence remains limited (16). Future studies should compare conventional paresthesia-based SCS with newer stimulation paradigms and assess outcomes beyond pain intensity, including prosthesis use, physical function, quality of life, sleep, opioid consumption, and patient global impression of change.

This study has several limitations. First, its retrospective, single-center design and small sample size reduced statistical power and increased the potential for selection bias. Second, the cohort was heterogeneous in terms of amputation etiology, pain location, lead type, and follow-up duration. Third, pain outcomes were assessed primarily using the NRS, whereas multidimensional outcomes, including physical function, prosthesis use, psychological status, sleep quality, quality of life, and analgesic consumption, were not systematically evaluated. Fourth, stimulation parameters and programming adjustments were not standardized over time. Fifth, the *post hoc* analysis of patients who retained their devices at the last available follow-up was subject to survivor bias. In addition, minor adverse events or events managed at other institutions may have been underascertained because of the retrospective study design. Finally, the regression analyses were exploratory because of the small number of outcome events, particularly device explantations, and the resulting estimates may have been unstable.

## 5 Conclusions

In this retrospective cohort, SCS provided substantial short-term pain relief in selected patients with refractory PLP, and most patients who underwent permanent implantation retained their devices at the last available follow-up. However, the magnitude of pain relief diminished over time, and 28% of permanently implanted patients underwent device explantation. These findings suggest that SCS may benefit selected patients with refractory PLP, although long-term outcomes vary and patients require continued follow-up and individualized device management.

## Data Availability

The raw data supporting the conclusions of this article will be made available by the authors, without undue reservation.
